# Association of time-to-treatment with prognosis in pneumocystis pneumonia among immunocompromised patients without HIV infection: a multi-center, retrospective observational cohort study

**DOI:** 10.1186/s12879-025-10933-3

**Published:** 2025-04-15

**Authors:** Haruka Fujioka, Hiroki Matsui, Yuya Homma, Tatsuya Nagai, Ayumu Otsuki, Hiroyuki Ito, Shinichiro Ohmura, Toshiaki Miyamoto, Daisuke Shichi, Watari Tomohisa, Yoshihito Otsuka, Kei Nakashima

**Affiliations:** 1 Department of Pulmonology, Kameda Medical Center, Chiba, Japan; 2 Department of Clinical Epidemiology and Health Economics, School of Public Health, The University of Tokyo, Tokyo, Japan; 3 Department of Clinical Research Support Office, Kameda Medical Center, Chiba, Japan; 4 Department of Rheumatology, Seirei Hamamatsu General Hospital, Aichi, Japan; 5 Department of Infection and Rheumatology, Seirei Mikatahara General Hospital, Shizuoka, Japan; 6 Department of Clinical Laboratory, Kameda Medical Center, Chiba, Japan

**Keywords:** Non-human immunodeficiency virus-infected patient, Pneumocystis pneumonia, Prognosis, Time-to-treatment

## Abstract

**Background:**

*Pneumocystis jirovecii* pneumonia (PCP) in non-human immunodeficiency virus (HIV) patients is associated with high morbidity and mortality. Although prior studies have linked delayed treatment to worse outcomes, they are often limited by small sample sizes and inadequate adjustment for confounders. Therefore, we evaluated whether early treatment after hospital admission improves mortality in non-HIV PCP, adjusting for patient characteristics.

**Methods:**

This multi-center, retrospective, observational cohort study included non-HIV PCP patients treated between January 2006 and March 2021 at three institutions. Participants were divided into the early treatment (initiated within 2 days) and late treatment (initiated between days 3 and 7) groups. The primary endpoint was 30-day mortality, and the secondary endpoints were 180-day mortality. Propensity score weighting was used to adjust for patient background.

**Results:**

Ninety-four patients in the early treatment group and 43 in the late treatment group were evaluated. The average time-to-treatment for the early and late treatment groups was 0.13 days and 3.63 days, respectively. After adjusting for patient characteristics, there were no significant differences in 30-day mortality (14.0% vs. 8.2%, *p* = 0.307) or 180-day mortality (21.5% vs. 17.7%, *p* = 0.600) between the early and late treatment groups. In a subgroup analysis of cases requiring oxygen supplementation, 30-day and 180-day mortality also showed no significant differences between the two groups.

**Conclusion:**

This study emphasizes the importance of accurate diagnosis and tailored management based on disease severity rather than immediate empirical treatment, as early treatment initiation was not significantly associated with 30-day or 180-day mortality in non-HIV PCP.

**Supplementary Information:**

The online version contains supplementary material available at 10.1186/s12879-025-10933-3.

## Background

*Pneumocystis jirovecii* pneumonia (PCP) is an opportunistic infection of patients with the human immunodeficiency virus (HIV)-infection-induced immunosuppression [1, 2]. PCP confers serious morbidity and mortality in immunocompromised patients, although the availability of highly active antiretroviral therapy has decreased the prevalence of acquired immunodeficiency syndrome in recent years [1, 3]. However, the number of patients with PCP but without HIV (non-HIV PCP) has increased as the number of those receiving anti-tumor chemotherapeutic agents, immunosuppressive therapy, and organ transplantation has been increasing [1, 4]. The mortality during the initial infection in patients with HIV PCP and non-HIV PCP is 10–20% and 30–60%, respectively [1, 2].

Prognostic factors for non-HIV PCP include several factors, such as advanced age, elevated lactate dehydrogenase (LDH) levels, low albumin, respiratory failure, hematological malignancies, increased blood urea nitrogen, concurrent bacteremia, and pre-existing lung disease [5, 6]. Furthermore, a delay in treatment is a prognostic factor for non-HIV PCP associated with worse outcomes [5, 7–10]. However, previous research on early treatment of non-HIV PCP had limitations, including a small sample and inadequate adjustment for confounding factors. For instance, a multi-center study conducted in France included both HIV PCP and non-HIV PCP cases, which made it difficult to accurately evaluate whether time-to-treatment contributes to mortality in non-HIV PCP [7]. Furthermore, another prior study noted the survival-related importance of early diagnosis and treatment initiation for non-HIV PCP patients; however, the study was limited by a small sample size of 23 patients and a retrospective single-center study design that impacted the scope and generalizability [10]. Sulfamethoxazole-trimethoprim (SMX/TMP), the first-line treatment for PCP, carries significant adverse effects in patients with non-HIV, including rash, cytopenia, and electrolyte abnormalities [11]. As a result, in patients with non-HIV PCP, the timing of treatment initiation becomes a critical clinical decision, balancing efficacy and toxicity.

Therefore, in this multi-center study, we evaluated whether an early interval from hospital admission to treatment, with adjustment for confounding factors, improved the prognosis of non-HIV PCP.

## Methods

From January 2006 to March 2021, we conducted a multi-center retrospective observational cohort study to assess non-HIV PCP patients at the Kameda Medical Center, Seirei Hamamatsu General Hospital, and Seirei Mikatahara General Hospital (registry named: RE-VISION-PCP: Registry to Provide New Evidence and Insights for the Management of Pneumocystis Pneumonia in Non-HIV-infected Patients).

The inclusion criteria were non-HIV patients aged 20 years who met the diagnostic criteria for PCP. Based on previous studies and a diagnostic guideline of non-HIV PCP [11–13], we defined the diagnosis criteria of PCP as all of the following: (1) host factor: possible immunocompromised status other than HIV; 2) clinical criteria: clinical symptoms consistent with those of PCP (dyspnoea, cough, fever, and hypoxemia) and chest radiograph or chest CT scan findings compatible with PCP (including bilateral or diffuse ground-glass opacities); and (3) microbial criteria: *Pneumocystis* species detected in respiratory specimens, such as sputum or bronchoalveolar lavage fluid, through conventional staining (Grocott methenamine silver or Diff-Quick staining) or DNA testing (loop-mediated isothermal amplification or polymerase chain reaction), or elevated β-D-glucan concentrations with an appropriate response to standard therapy for PCP. Serum β-D-glucan concentrations were measured using either the β-D-glucan test kit (Wako Pure Chemical Industries) or the FUNGITEC G test MKII (Nissui Pharmaceutical). Elevated β-D-glucan levels were defined as concentrations > 5 pg/mL (β-D-glucan test; Wako assay) or > 20 pg/mL (FUNGITEC G-test KM assay) [14–16]. The exclusion criteria for the study were as follows: (1) patients for whom treatment had not been initiated and (2) patients who did not initiate treatment within 7 days. In previous studies, treatment of non-HIV PCP patients has typically commenced within 7 days from the day of hospitalization [7, 17], and symptoms may worsen within the first 7 days of onset [1]. Thus, in this study, it was necessary to compare the timing of treatment initiation and prognosis in cases wherein PCP treatment was appropriately administered. Therefore, patients who did not initiate treatment within 7 days of hospitalization were excluded.

This study received ethical approval and a review of the research proposal from the Institutional Review Boards of the participating institutions, including Kameda Medical Center (#21–069–230801), Seirei Hamamatsu Hospital (#3584), and Seirei Mikatahara Hospital (#21–58). This study was conducted in accordance with the principles of the Declaration of Helsinki. As this study was retrospective and the patient information was anonymized, the requirement for written informed consent was waived, and information regarding the right to opt out of the study was provided.

### Exposure definition

In this study, time to treatment was defined as the duration (days) from the day of hospitalization (Day 1) to treatment initiation. The cases were divided into two groups: the ‘early treatment group,’ which initiated treatment within 2 days, and the ‘late treatment group’, which started treatment between days 3 and 7. This classification was based on the median time to treatment initiation for non-HIV PCP reported in previous studies, which is approximately 2 to 3 days [7, 17, 18]. Additionally, international guidelines recommend initiating treatment promptly upon suspicion of non-HIV PCP [19], further supporting the rationale for defining early treatment as initiation within the first 2 days of hospitalization.

### Setting and treatment strategy

Based on the international treatment guideline [20], the primary treatment of choice for PCP was SMX/TMP and in cases with treatment difficulties such as allergic reactions or side effects, treatment with alternative therapeutic agents was continued. Atovaquone and pentamidine were used as second-line treatments. Steroids were administered, especially in patients with respiratory failure, as per the treatment protocol for HIV PCP [21].

### Data collection

The participants’ characteristics data included age, sex, weight, hospital, underlying diseases, and immunosuppressive agents used at the time of presentation, such as glucocorticoids (including daily dosage converted to prednisolone equivalent), immunosuppressants, biologic immunosuppressive drugs, and antineoplastic drugs. Glucocorticoid use at onset was defined as the oral administration of glucocorticoids, regardless of dosage, while excluding inhaled steroids. Recorded laboratory parameters included white blood cell count, neutrophil count, lymphocyte count, hemoglobin, platelet count, albumin, LDH, sodium, potassium, and creatinine, as well as creatinine clearance. Clinical characteristics included the presence of altered consciousness, presence of hypotension, and respiratory status (without oxygen, administration of oxygen, or mechanical ventilation). Treatment-related data included the initial therapeutic agents, dosage of SMX/TMP, total treatment duration, adjunctive steroid therapy, and duration (days) from hospitalization to treatment initiation. The dosage of SMX/TMP was adjusted using a correction factor based on creatinine clearance (CrCl), as dose modification is necessary according to the severity of renal dysfunction [22]. CrCl was estimated using the Cockcroft-Gault equation [23]. The correction factors applied were as follows: 1.0 for CrCl > 50 mL/min, 1.5 for CrCl 30–50 mL/min, 2.0 for CrCl 15–30 mL/min, and 3.0 for CrCl < 15 mL/min or patients undergoing hemodialysis [22]. The use of glucocorticoids was defined as an increase in the glucocorticoid dosage compared to the dose administered prior to PCP onset. Pulse therapy with glucocorticoids was defined as the administration of ≥ 500 mg methylprednisolone for three consecutive days or more. Mortality was assessed at 30 days and 180 days. As supplementary information, data on grade 3 or higher adverse events, as defined by the Common Terminology Criteria for Adverse Events (CTCAE) Version 5.0, associated with SMX/TMP administration in treated patients were also collected [24]. However, information on the adverse effects of other drugs, such as atovaquone and pentamidine, was not originally included in this study.

### Outcomes

The primary endpoint was the 30-day mortality rate, and the secondary endpoint was the 180-day mortality rate.

### Statistical analysis

As this was a retrospective observational study, a sample-size calculation was not performed. Although one case had missing data for neutrophil and lymphocyte counts, these variables were not used for covariate adjustment. Continuous and categorical variables are presented as mean ± standard deviation and frequency (proportion), respectively. Intergroup comparisons were conducted using the Student’s *t*-test for continuous variables and the chi-square test for categorical variables. The baseline characteristics of the patients’ backgrounds and severity were compared between the early and late treatment groups. Balance in terms of baseline characteristics between the early and late treatment groups was evaluated using the standardized mean difference (SMD). Generally, a group is considered balanced when the SMD is < 0.1 [25]. To obtain a risk-adjusted cohort, inverse probability of treatment weighting (IPTW) with propensity scores was applied [25]. Propensity scores were calculated using logistic regression with exposure as the dependent variable and age, sex, hospital, prior malignancies, serum albumin levels, LDH levels, creatinine clearance, and respiratory status. These explanatory variables were chosen based on the results of hospital clustering and prognostic factors for non-HIV PCP [5, 19]. After IPTW, each patient was assigned a weight, resulting in an adjusted cohort size that may differ from the original sample size and may contain decimal values. The adjusted cohort size reflects the reweighted sample rather than the actual number of individuals. Subsequently, patient outcomes were compared between the early and late treatment groups. In addition, we conducted a subgroup analysis restricted to patients requiring oxygen supplementation to compare outcomes between the early and late treatment groups in severe cases. The significance level was set at *p* < 0.05.

### Sensitivity analysis

We conducted a sensitivity analysis by truncating extreme weights. Specifically, following a previous study, we truncated the IPTW at the 1st and 99th percentiles [26]. Using these trimmed weights, we repeated the analysis in the overall population as well as in the subgroup requiring oxygen supplementation.

## Results

**Fig. 1:**
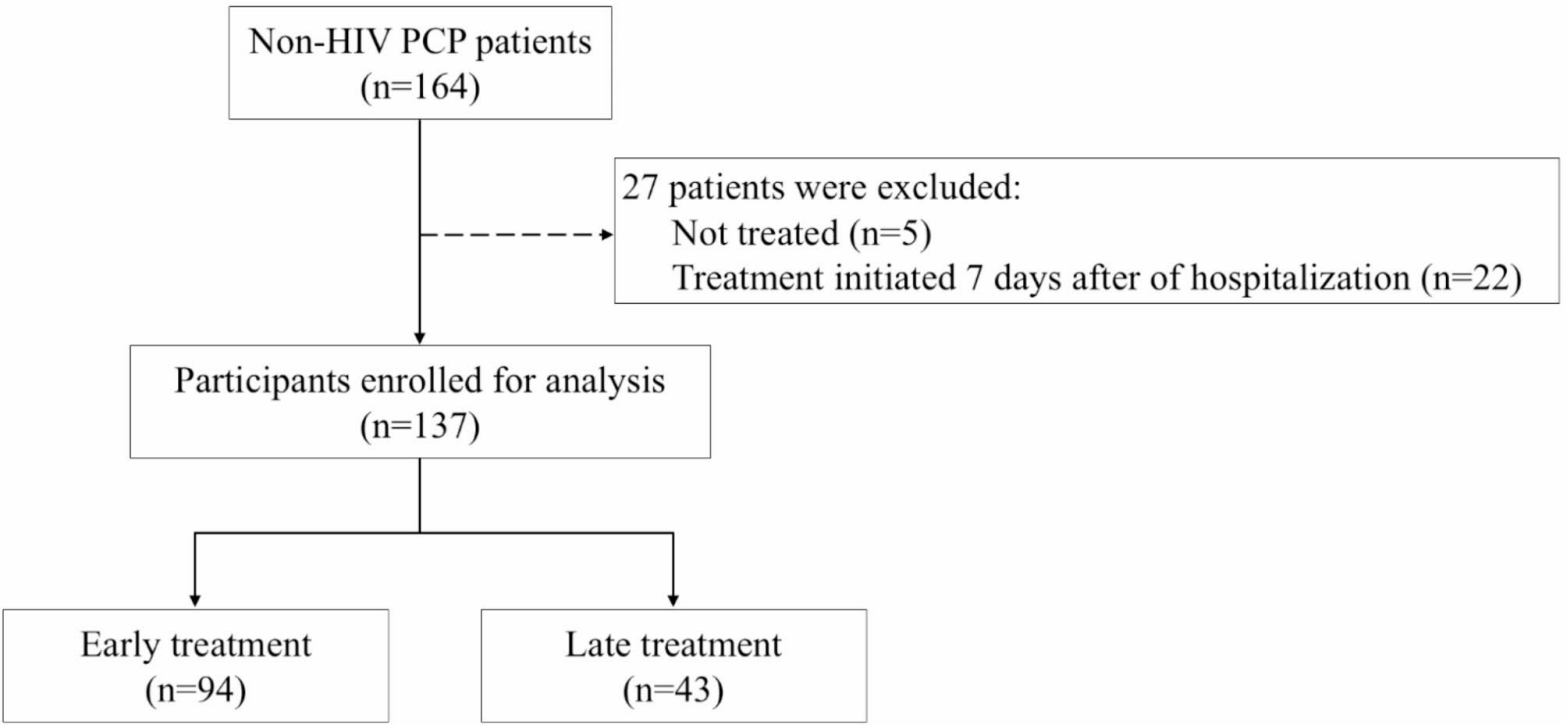
Patient selection flowchart HIV, human immunodeficiency virus; PCP, *Pneumocystis jirovecii* pneumonia

Between 1 January 2006 and 31 March 2021, 164 patients were diagnosed with non-HIV PCP after being admitted to one of the three hospitals. Five individuals were excluded because they did not receive treatment. An additional 22 patients were excluded because they did not commence treatment within 7 days of hospitalization. Ultimately, 137 patients began treatment for non-HIV PCP within 7 days of hospital admission. All the eligible patients were treated with anti-PCP medications. The early and late treatment groups comprised 94 and 43 patients, respectively (Fig. 1).

Patient characteristics are presented in Table 1. Among the 137 individuals in the study, the average time-to-treatment for the early treatment and late treatment groups was 0.13 (± 0.34) and 3.63 (± 1.45) days, respectively. Patient backgrounds before and after conducting weighted propensity score matching are described in ‘Before adjustment’ and ‘After adjustment,’ respectively. As expected with IPTW, the adjusted cohort size appears larger than the original sample size due to the assigned weights and included decimal values, reflecting the fractional nature of the population count, which helps balance covariates across groups. Before adjustment, an SMD greater than 0.1 was observed for several variables, including female sex, weight, hospital, underlying diseases, immunosuppressive agents used, hemoglobin, albumin, LDH, serum sodium, serum potassium, creatinine clearance, altered consciousness, hypotension, and respiratory status. Details of immunosuppressants and biologic immunosuppressive drugs in unadjusted cohort are shown in Supplementary Table 1. Regarding underlying diseases, a higher proportion of patients with malignancy was observed in the late treatment group. After adjustment, the SMD was reduced to less than 0.1 for most adjustment variables, such as age, sex, hospital, malignancy, albumin, LDH, and creatinine clearance. However, the SMD for respiratory status remained above 0.1, indicating residual imbalance for this variable.

**Table 1:** Baseline characteristics of the study population

	Unadjusted patient cohort			Adjusted patient cohort		
	Early treatment group, *n* = 94	Late treatment group, *n* = 43	SMD	Early treatment group, *n* = 136.24	Late treatment group, *n* = 135.81	SMD
Age, years	71.03 (10.43)	70.42 (8.59)	0.064	70.86 (10.47)	70.43 (8.18)	0.045
Female	54 (57.4)	22 (51.2)	0.126	74.1 (54.4)	72.3 (53.2)	0.023
Weight, kg	52.00 (11.08)	54.70 (12.13)	0.232	52.47 (10.94)	53.35 (12.09)	0.077
Hospital						
Kameda Medical Center	49 (52.1)	25 (58.1)	0.414	72.8 (53.5)	67.9 (50.0)	0.083
Seirei Hamamatsu General Hospital	37 (39.4)	10 (23.3)		47.6 (34.9)	52.9 (38.9)	
Seirei Mikatahara General Hospital	8 (8.5)	8 (18.6)		15.8 (11.6)	15.0 (11.0)	
Underlying diseases						
Malignancy	14 (14.9)	11 (25.6)	0.268	24.8 (18.2)	24.2 (17.8)	0.009
Hematologic malignancy	6 (6.4)	5 (11.6)	0.184	10.1 (7.4)	11.8 (8.7)	0.047
Solid tumor	9 (9.6)	7 (16.3)	0.201	16.2 (11.9)	14.1 (10.4)	0.046
Connective tissue disease	80 (85.1)	28 (65.1)	0.475	112.9 (82.9)	101.5 (74.8)	0.199
Immunosuppressive agents used						
Glucocorticoids	58 (61.7)	30 (69.8)	0.171	84.3 (61.9)	90.5 (66.6)	0.098
Glucocorticoid dose converted to prednisolone equivalent, mg/day	12.26 (12.87)	13.76 (13.53)		12.03 (12.92)	13.19 (13.26)	
Immunosuppressant	70 (74.5)	23 (53.5)	0.448	99.2 (72.8)	88.6 (65.2)	0.164
Biologic immunosuppressive drugs	28 (29.8)	10 (23.3)	0.148	37.7 (27.7)	39.8 (29.3)	0.035
Antineoplastic drugs	5 (5.3)	7 (16.3)	0.359	9.0 (6.6)	15.5 (11.4)	0.169
Blood test findings						
White blood cell count, /µL	8724.68 (4808.22)	8853.95 (5067.86)	0.026	8979.85 (5131.52)	9313.04 (5624.81)	0.062
Neutrophil count ^a^, /µL	6579.19 (3295.39)	6799.71 (3697.74)	0.063	6653.96 (3279.26)	6885.67 (3678.86)	0.066
Lymphocyte count ^a^, /µL	1146.43 (1157.49)	1173.61 (1376.02)	0.021	1136.16 (1097.80)	1383.34 (1817.69)	0.165
Hemoglobin, g/dL	11.74 (1.96)	11.36 (1.74)	0.202	11.75 (1.94)	11.38 (1.72)	0.197
Platelet count, ×10⁴/µL	21.39 (10.11)	22.11 (10.12)	0.072	21.39 (9.98)	22.65 (9.52)	0.129
Albumin, g/dL	3.07 (0.60)	2.94 (0.63)	0.218	3.04 (0.60)	3.07 (0.64)	0.049
Lactate dehydrogenase, IU/L	400.01 (151.99)	417.88 (164.33)	0.113	408.42 (161.70)	411.57 (151.22)	0.020
Serum sodium, mEq/L	137.39 (3.68)	137.86 (5.24)	0.103	137.18 (3.62)	137.39 (4.72)	0.048
Serum potassium, mEq/L	4.29 (0.49)	4.07 (0.45)	0.463	4.29 (0.49)	4.15 (0.40)	0.302
Creatinine, mg/dL	1.12 (1.30)	1.10 (1.82)	0.011	1.11 (1.31)	1.18 (1.91)	0.046
Creatinine clearance, mL/min	57.85 (26.47)	64.31 (29.30)	0.231	59.24 (26.61)	58.69 (27.05)	0.021
Altered consciousness	1 (1.1)	0 (0.0)	0.147	2.2 (1.6)	0.0 (0.0)	0.180
Hypotension (systolic pressure < 90 mmHg)	3 (3.2)	0 (0.0)	0.257	3.0 (2.2)	0.0 (0.0)	0.212
Respiratory status			0.251			0.138
Without oxygen	59 (62.8)	23 (53.5)		82.9 (60.8)	87.5 (64.4)	
Administration of oxygen	34 (36.2)	20 (46.5)		52.4 (38.4)	48.3 (35.6)	
1–4 L/min	23 (24.5)	10 (23.3)		33.5 (24.6)	23.9 (17.6)	
5–10 L/min	4 (4.3)	6 (14.0)		5.9 (4.3)	12.5 (9.2)	
11–15 L/min	7 (7.4)	4 (9.3)		12.9 (9.5)	11.9 (8.8)	
Mechanical ventilation	1 (1.1)	0 (0.0)		1.0 (0.7)	0.0 (0.0)	
Time from admission to treatment initiation	0.13 (0.34)	3.63 (1.45)	3.331	0.14 (0.35)	3.55 (1.50)	3.142
Initial therapeutic agents			0.069			0.168
SMX/TMP	85 (90.4)	38 (88.4)		124.3 (91.2)	116.7 (85.9)	
Dose of SMX/TMP administration, mg/kg/d	17.71 (13.55)	15.64 (9.43)		17.10 (12.59)	17.67 (11.00)	
Atovaquone	7 (7.4)	4 (9.3)		9.4 (6.9)	15.5 (11.4)	
Pentamidine	2 (2.1)	1 (2.3)		2.6 (1.9)	3.5 (2.6)	
Total treatment duration	17.36 (6.91)	19.86 (12.30)	0.251	16.96 (7.08)	18.91 (11.15)	0.209
Adjunctive glucocorticoid therapy						
None	10 (10.6)	7 (16.3)	0.166	13.6 (10.0)	21.1 (15.5)	0.168
Yes (mild-to-moderate dose)	29 (30.9)	18 (41.9)	0.230	43.8 (32.2)	51.4 (37.8)	0.119
Yes (steroid pulse therapy)	55 (58.5)	18 (41.9)	0.338	78.8 (57.9)	63.3 (46.6)	0.227

Continuous and categorical variables are expressed as the mean ± standard deviation and number (%), respectively

Adjusted totals and numbers represent the effective sample size and event counts after inverse probability weighting, where each patient contributes a fractional weight rather than a discrete count

^a^ Missing data were observed in one case each for neutrophil and lymphocyte counts

SMD, standardized mean difference: SMX/TMP, Trimethoprim-sulfamethoxazole

Data on the primary and secondary endpoints are presented in Table 2. Before adjustment, the 30-day mortality rates were 11.7% and 11.6% in the early and late treatment groups, respectively (*p* = 1.000). At 180 days, the mortality rates were 19.1% and 25.6% in the early and late treatment groups, respectively (*p* = 0.529). After adjustment, there were no significant differences in the 30-day mortality (14.0% vs. 8.2%, *p* = 0.307) and 180-day mortality rates (21.5% vs. 17.7%, *p* = 0.600) between the early and late treatment groups. Data on CTCAE grade 3 or higher adverse events associated with SMX/TMP administration in the unadjusted cohort of treated patients are presented in Supplementary Table 2. Rash, hyponatremia, hyperkalemia, and nausea were the most frequently observed adverse effects, with approximately half of the patients experiencing CTCAE grade 3 or higher toxicity.

**Table 2:** Summary of clinical outcomes

	Unadjusted patient cohort			Adjusted patient cohort		
	Early treatment group, *n* = 94	Late treatment group, *n* = 43	*P*	Early treatment group, *n* = 136.2	Late treatment group, *n* = 135.8	*P*
Primary endpoint						
30-day mortality	11 (11.7)	5 (11.6)	1.000	19.0 (14.0)	11.1 (8.2)	0.307
Secondary						
180-day mortality	18 (19.1)	11 (25.6)	0.529	29.3 (21.5)	24.1 (17.7)	0.600

Categorical variables are expressed as number (%)

Adjusted totals and numbers represent the effective sample size and event counts after inverse probability weighting, where each patient contributes a fractional weight rather than a discrete count

Next, we performed a subgroup analysis restricted to patients requiring oxygen supplementation. The early treatment and late treatment groups included 35 and 20 patients, respectively. Patient characteristics of the subgroup are presented in Table 3. Before adjustment, an SMD greater than 0.1 was identified for several variables, including female sex, weight, hospital, underlying diseases, immunosuppressant, biologic immunosuppressive drugs, antineoplastic drugs, white blood cell count, neutrophil count, lymphocyte count, hemoglobin, albumin, LDH, serum sodium, serum potassium, creatinine clearance, hypotension, and respiratory status. Following adjustment, the SMD was reduced to below 0.1 for key variables such as age, malignancy, albumin, and LDH, indicating improved balance in these factors. The data on primary and secondary endpoints derived from the subgroup analysis are shown in Table 4. After weighting, the 30-day mortality (24.8% vs. 17.6%, *p* = 0.577) and the 180-day mortality rates (33.9% vs. 40.0%, *p* = 0.691) showed no significant difference between the early and late treatment groups.

**Table 3:** Baseline characteristics of the subgroup requiring oxygen supplementation

	Unadjusted patient cohort			Adjusted patient cohort		
	Early treatment group, *n* = 35	Late treatment group, *n* = 20	SMD	Early treatment group, *n* = 52.38	Late treatment group, *n* = 49.63	SMD
Age, years	71.89 (9.48)	72.60 (8.04)	0.081	72.12 (9.55)	72.54 (7.43)	0.050
Female	16 (45.7)	11 (55.0)	0.187	23.8 (45.4)	26.9 (54.1)	0.175
Weight, kg	53.20 (10.92)	55.25 (11.31)	0.184	53.15 (10.79)	54.80 (11.39)	0.149
Hospital						
Kameda Medical Center	27 (77.1)	17 (85.0)	0.469	42.0 (80.2)	38.2 (76.9)	0.207
Seirei Hamamatsu General Hospital	8 (22.9)	2 (10.0)		10.4 (19.8)	10.5 (21.1)	
Seirei Mikatahara General Hospital	0 (0.0)	1 (5.0)		0.0 (0.0)	1.0 (2.0)	
Underlying diseases						
Malignancy	8 (22.9)	8 (40.0)	0.376	15.4 (29.4)	14.5 (29.2)	0.004
Hematologic malignancy	4 (11.4)	3 (15.0)	0.106	7.4 (14.1)	4.9 (9.9)	0.131
Solid tumor	4 (11.4)	6 (30.0)	0.471	8.0 (15.3)	11.4 (22.9)	0.196
Connective tissue disease	28 (80.0)	9 (45.0)	0.775	39.4 (75.2)	28.4 (57.2)	0.387
Immunosuppressive agents used						
Glucocorticoid	26 (74.3)	15 (75.0)	0.016	37.9 (72.3)	42.5 (85.7)	0.333
Glucocorticoid dose converted to prednisolone equivalent	12.60 (14.71)	13.54 (14.66)		11.81 (13.57)	14.60 (14.73)	
Immunosuppressant	23 (65.7)	5 (25.0)	0.896	32.8 (62.5)	17.6 (35.5)	0.561
Biologic immunosuppressive drugs	5 (14.3)	1 (5.0)	0.319	7.1 (13.6)	2.0 (4.0)	0.343
Antineoplastic drugs	4 (11.4)	5 (25.0)	0.357	7.9 (15.0)	9.6 (19.3)	0.114
Blood test findings						
White blood cell count, /µL	9670.29 (5173.56)	8754.50 (3879.33)	0.200	10799.59 (6781.78)	8975.83 (3767.54)	0.332
Neutrophil count^a^, /µL	7486.90 (3161.21)	7139.82 (3078.48)	0.111	7676.17 (3285.66)	7374.73 (3071.01)	0.095
Lymphocyte count^a^, /µL	1015.71 (716.18)	921.35 (556.70)	0.147	1072.61 (743.18)	967.53 (646.42)	0.151
Hemoglobin, g/dL	11.91 (2.23)	11.70 (1.23)	0.114	11.79 (2.12)	11.91 (1.24)	0.068
Platelet count (×10⁴/µL)	22.59 (11.54)	21.76 (9.54)	0.078	22.35 (11.30)	22.22 (10.34)	0.012
Albumin, g/dL	2.80 (0.54)	2.68 (0.56)	0.216	2.77 (0.53)	2.73 (0.52)	0.072
Lactate dehydrogenase, IU/L	418.34 (166.91)	467.85 (188.61)	0.278	443.49 (189.98)	450.92 (166.73)	0.042
Serum sodium, mEq/L	137.26 (4.07)	139.50 (6.53)	0.412	136.96 (3.91)	139.82 (5.30)	0.615
Serum potassium, mEq/L	4.22 (0.50)	3.98 (0.50)	0.466	4.22 (0.53)	4.13 (0.44)	0.186
Creatinine, mg/dL	1.34 (1.76)	0.78 (0.27)	0.444	1.19 (1.59)	0.80 (0.21)	0.341
Creatinine clearance, mL/min	54.60 (26.19)	68.98 (28.82)	0.522	58.09 (26.20)	62.82 (22.99)	0.192
Altered consciousness	0 (0.0)	0 (0.0)	< 0.001	0 (0.0)	0 (0.0)	< 0.001
Hypotension (systolic pressure < 90 mmHg)	2 (5.7)	0 (0.0)	0.348	2.0 (3.8)	0.0 (0.0)	0.282
Respiratory status			0.243			0.197
Administration of oxygen	34 (97.1)	20 (100.0)		51.4 (98.1)	49.6 (100.0)	
1–4 L/min	23 (65.7)	10 (50.0)		31.8 (60.8)	21.5 (43.4)	
5–10 L/min	4 (11.4)	6 (30.0)		5.5 (10.4)	14.5 (29.3)	
11–15 L/min	7 (20.0)	4 (20.0)		14.1 (26.9)	13.6 (27.4)	
Mechanical ventilation	1 (2.9)	0 (0.0)		1.0 (1.9)	0.0 (0.0)	
Time from admission to treatment initiation	0.11 (0.32)	3.10 (1.12)	3.625	0.11 (0.32)	2.91 (1.09)	3.475
Initial therapeutic agents			0.032			0.191
SMX/TMP	33 (94.3)	19 (95.0)		49.3 (94.2)	44.1 (89.0)	
Dose of SMX/TMP administration, mg/kg/d	16.03 (7.21)	12.49 (6.74)		15.49 (6.87)	13.74 (6.51)	
Atovaquone	2 (5.7)	1 (5.0)		3.0 (5.8)	5.5 (11.0)	
Total treatment duration	16.71 (8.65)	20.15 (11.23)	0.343	16.09 (8.64)	21.58 (14.33)	0.464
Adjunctive glucocorticoid therapy						
None	1 (2.9)	2 (10.0)	0.294	1.1 (2.0)	3.9 (7.8)	0.270
Yes (mild-to-moderate dose)	15 (42.9)	11 (55.0)	0.245	22.9 (43.7)	27.2 (54.8)	0.223
Yes (steroid pulse therapy)	19 (54.3)	7 (35.0)	0.395	28.4 (54.3)	18.6 (37.4)	0.344

Continuous and categorical variables are expressed as the mean ± standard deviation and number (%), respectively

Adjusted totals and numbers represent the effective sample size and event counts after inverse probability weighting, where each patient contributes a fractional weight rather than a discrete count

^a^ Missing data were observed in one case each for neutrophil and lymphocyte counts

SMD, standardized mean difference: SMX/TMP, Trimethoprim-sulfamethoxazole

**Table 4:** Clinical outcomes of the subgroup requiring oxygen supplementation

Survival	Unadjusted patient cohort			Adjusted patient cohort		
	Early treatment group, *n* = 35	Late treatment group, *n* = 20	*P*	Early treatment group, *n* = 52.4	Late treatment group, *n* = 49.6	*P*
Primary endpoint						
30-day mortality	7 (20.0)	4 (20.0)	1.000	13.0 (24.8)	8.7 (17.6)	0.577
Secondary endpoint						
180-day mortality	11 (31.4)	9 (45.0)	0.475	17.7 (33.9)	19.9 (40.0)	0.691

Categorical variables are expressed as number (%)

Adjusted totals and numbers represent the effective sample size and event counts after inverse probability weighting, where each patient contributes a fractional weight rather than a discrete count

Finally, we conducted a sensitivity analysis using IPTW with truncation at the 1st and 99th percentiles. Patient characteristics for the overall population after trimming are shown in Supplementary Table 3, while primary and secondary endpoints are summarized in Supplementary Table 4. The results remained largely unchanged, showing no significant differences in the 30-day mortality and 180 mortality rates between the early and late treatment groups. Similarly, the results of the sensitivity analysis in the subgroup of patients requiring oxygen supplementation are presented in Supplementary Table 5 (patient characteristics) and Supplementary Table 6 (primary and secondary endpoints). Again, no significant differences were observed in the 30-day mortality and 180-day mortality rates between the treatment groups.

## Discussion

In this study, no significant differences were observed in the 30-day and 180-day mortality between the early and late treatment groups in the management of non-HIV PCP, even among the subgroup requiring oxygen. Additionally, similar results were obtained in the sensitivity analysis using IPTW with truncation at the 1st and 99th percentiles. Utilizing data from the largest multicenter cohort to date to analyze the effects of early versus late treatment, this study demonstrated that treatment delay did not impact short-term mortality outcomes in non-HIV PCP.

Similar to our study, other research has reported no prognostic difference between early treatment and later treatment. Ko et al. investigated the interval from emergency room admission to the initiation of PCP treatment in 51 patients with non-HIV PCP and respiratory failure and found no association between the time to treatment initiation and increased mortality in non-HIV PCP [18]. Our study, which focused on cases of respiratory failure (patients requiring oxygen) and comprised approximately 50 cases, demonstrated a high level of reliability consistent with Ko et al.‘s research. Conversely, two other retrospective studies (Asai et al. and Overgaard et al.) on non-HIV PCP patients indicated that the time from hospital admission to treatment initiation was associated with in-hospital mortality [9, 10]. However, these studies had limitations, including small sample sizes (ranging from 23 to 48 cases) and the use of univariate analysis without adjusting for prognostic factors related to mortality. Li et al. reported that the longer time from hospitalization to treatment initiation in non-HIV PCP compared to HIV PCP might explain the higher mortality in non-HIV PCP. However, other factors, including age and pneumonia severity index differences, may have also contributed [8]. Without adjusting for prognostic factors, it is unclear whether early treatment directly impacts prognosis. Additionally, the median time to treatment in the late group of our study was 3.63 days, whereas in the studies by Asai et al., Li et al., and Overgaard et al., the median time to treatment initiation in the mortality groups was between 8.67 and 15 days [8–10], which may have contributed to the differing results.

In this study, we observed a higher percentages of patients with malignancies in the late treatment group within the unadjusted cohort. This difference was adequately balanced in our adjusted analysis, where malignancy was included as a variable in the propensity score weighting. One possible explanation for this initial imbalance is that in patients with malignancies presenting with respiratory symptoms or pulmonary infiltrates, the differential diagnosis tends to be broader, including disease progression, drug-induced pneumonitis, various infections, and PCP [27]. Furthermore, oncologists or hematologists, who primarily manage these patients, may have limited experience with diagnosing and treating PCP, potentially contributing to delays in initiating appropriate therapy until referral to pulmonologists occurs.

SMX/TMP, the first-line treatment for non-HIV PCP, is a therapeutic option that is associated with numerous side effects [11]. In our study, approximately half of the patients who received SMX/TMP experienced CTCAE grade 3 or higher adverse events. Therefore, it is crucial to diagnose and treat non-HIV PCP appropriately rather than empirically prescribing this treatment. Prognostic factors, such as age, LDH levels, respiratory failure, malignancy, and low albumin levels, have been reported, and these factors are associated with an increased mortality rate [5, 19]. Thus, it is important to assess the disease severity in patients based on these factors and to comprehensively consider patient management.

This study includes diverse underlying conditions and has undergone sufficient adjustment for patient backgrounds, thereby presenting more accurate statistical results. Subgroup analyses based on severity were conducted to enhance external validity. However, larger prospective randomized controlled trials or cohort studies are required in the future because the study was small scale and limited severe cases.

Nevertheless, this study has some limitations. First, this was a retrospective observational study, and despite efforts to align patient backgrounds between the early and late treatment groups, there was a potential for selection bias. It cannot be ruled out that cases with mild symptoms, where observation without treatment was possible, may have selectively been included in the late treatment group. However, in the subgroup analysis of severe cases requiring oxygen at the time of admission, no significant difference was observed between the early and late treatment groups. Therefore, we speculated that the bias in patient severity was not substantial. Second, though this study examined short-term mortality outcomes, it did not evaluate long-term functional outcomes (Quality Of Life, respiratory function, and Activities of Daily Living). Therefore, the potential impact of early treatment initiation on long-term functional outcomes remains to be evaluated. Future prospective observational studies evaluating long-term outcomes are essential. Third, the inclusion of patients with diverse immunosuppressive conditions, such as autoimmune diseases, solid malignancies, hematologic malignancies, and other etiologies, inevitably introduces certain heterogeneity, potentially contributing to bias in our results. While we acknowledge this as a limitation, it reflects the real-world complexity of immunosuppression, making it difficult to fully standardize patient characteristics in such studies. Fourth, this study aimed to examine the relationship between early treatment initiation and prognosis in patients where PCP treatment was appropriately administered, specifically within 7 days, based on the descriptive from the clinical course of patients with non-HIV PCP [7, 17]. Given the rapidly progressive nature of non-HIV PCP, immediate initiation of treatment upon clinical suspicion is recommended [19]. Thus, we defined “early treatment” as therapy initiated ideally within 1 to 2 days after hospitalization [7, 17], and “late treatment” as therapy initiated between 3 and 7 days after hospitalization, representing a clinically meaningful delay. Cases where treatment began more than 7 days post-hospitalization were excluded from analysis, as such delays reflect atypical clinical practice. Thus, our findings may not be generalizable to patients whose treatment started beyond 7 days after hospitalization.

## Conclusion

This study found no significant association between early treatment initiation and 30-day or 180-day mortality rates in non-HIV PCP patients compared to the late treatment initiation group. Given the considerable side effects associated with the treatment of non-HIV PCP, the results suggest that, rather than immediately resorting to empirical treatment, it is crucial to make an accurate diagnosis and implement management that is individualized based on the disease severity.

## Electronic supplementary material

Below is the link to the electronic supplementary material.

Supplementary Material 1

Supplementary Material 2

## Data Availability

Deta pertaining to this study will be available by the corresponding author upon reasonable request.

## Abbreviations

PCP: *Pneumocystis jirovecii* pneumonia
HIV: Human immunodeficiency virus
LDH: Lactate dehydrogenase
CTCAE: Common Terminology Criteria for Adverse Events
SMX/TMP: Sulfamethoxazole–trimethoprim
CrCl: Creatinine clearance
IPTW: Inverse probability of treatment weighting
SMD: The standardized mean difference
