# Supplementary material for: Association of time-to-treatment with prognosis in pneumocystis pneumonia among immunocompromised patients without HIV infection: a multi-center, retrospective observational cohort study
Source: BMC Infect Dis. 2025 Apr 15;25:531. doi: 10.1186/s12879-025-10933-3 (PMC12001674; doi:10.1186/s12879-025-10933-3)
Supplement: Supplementary file 1 — Supplementary Material 1 [file 12879_2025_10933_MOESM1_ESM.docx]

Supplementary table 1. Details of immunosuppressants and biologic immunosuppressive drugs

|  | Early treatment group, n = 94 | Late treatment group, n = 43 |
| --- | --- | --- |
| Immunosuppressant^a^ | n = 70 | n = 23 |
| Tacrolimus | 2/70 (2.9) | 1/23 (4.3) |
| Cyclosporine | 4/70 (5.7) | 1/23 (4.3) |
| Azathioprine | 2/70 (2.9) | 3/23 (13.0) |
| Cyclophosphamide | 2/70 (2.9) | 0/23 (0) |
| Methotrexate | 61/70 (87.1) | 17/23 (73.9) |
| Bucillamine | 3/70 (4.3) | 2/23 (8.7) |
| Others | 2/70 (2.9) | 3/23 (13.0) |
| Biologic immunosuppressive drugs | n = 28 | n = 10 |
| Adalimumab | 13/28 (46.4) | 5/10 (50.0) |
| Abatacept | 5/28 (17.9) | 1/10 (10.0) |
| Infliximab | 5/28 (17.9) | 1/10 (10.0)) |
| Etanercept | 4/28 (14.3) | 2/10 (20.0) |
| Certolizumab | 0/28 (0) | 1/10 (10.0) |
| Rituximab | 1/28 (3.6) | 0/10 (0) |

Data are presented as number/total number (%)

^a^ Owing to duplications, the total number of each column exceeded 70 in the early treatment group and 23 cases in the BL group

Supplementary table 2. Details of grade 3 or higher adverse events in the unadjusted cohort of patients treated with sulfamethoxazole–trimethoprim

|  | Early treatment group, n = 85 | Late treatment group, n = 38 |
| --- | --- | --- |
| Total | 42 (49.4) | 18 (47.4) |
| Skin rashes | 16 (18.8) | 4 (10.5) |
| Nausea | 10 (11.8) | 2 (5.3) |
| Leukopenia | 1 (1.2) | 0 (0.0) |
| Anemia | 0 (0.0) | 2 (5.3) |
| Thrombocytopenia | 3 (3.5) | 0 (0.0) |
| Increased ALT levels | 2 (2.4) | 3 (7.9) |
| Hyponatremia | 18 (21.2) | 9 (23.7) |
| Hyperkalemia | 6 (7.1) | 7 (18.4) |

Data are presented as number (%)

ALT, alanine aminotransferase

**Supplementary table 3.** Baseline characteristics of the overall population in the sensitivity analysis using inverse probability of treatment weighting with truncation at the 1st and 99th percentiles

|  | **Unadjusted patient cohort** | | | **Adjusted patient cohort** | | |
| --- | --- | --- | --- | --- | --- | --- |
|  | **Early treatment group, n = 94** | **Late treatment group, n =43** | **SMD** | **Early treatment group, n = 136.24** | **Late treatment group, n = 135.02** | **SMD** |
| Age, years | 71.03 (10.43) | 70.42 (8.59) | 0.064 | 70.86 (10.47) | 70.41 (8.19) | 0.048 |
| Female | 54 (57.4) | 22 (51.2) | 0.126 | 74.1 (54.4) | 72.3 (53.5) | 0.017 |
| Weight, kg | 52.00 (11.08) | 54.70 (12.13) | 0.232 | 52.47 (10.94) | 53.42 (12.09) | 0.083 |
| Hospital |  |  |  |  |  |  |
| Kameda Medical Center | 49 (52.1) | 25 (58.1) | 0.414 | 72.8 (53.5) | 67.9 (50.3) | 0.075 |
| Seirei Hamamatsu General Hospital | 37 (39.4) | 10 (23.3) |  | 47.6 (34.9) | 52.1 (38.6) |  |
| Seirei Mikatahara General Hospital | 8 (8.5) | 8 (18.6) |  | 15.8 (11.6) | 15.0 (11.1) |  |
| Underlying diseases |  |  |  |  |  |  |
| Malignancy | 14 (14.9) | 11 (25.6) | 0.268 | 24.8 (18.2) | 24.2 (17.9) | 0.006 |
| Hematologic malignancy | 6 (6.4) | 5 (11.6) | 0.184 | 10.1 (7.4) | 11.8 (8.7) | 0.049 |
| Solid tumor | 9 (9.6) | 7 (16.3) | 0.201 | 16.2 (11.9) | 14.1 (10.5) | 0.044 |
| Connective tissue disease | 80 (85.1) | 28 (65.1) | 0.475 | 112.9 (82.9) | 100.7 (74.6) | 0.203 |
| Immunosuppressive agents used |  |  |  |  |  |  |
| Glucocorticoids | 58 (61.7) | 30 (69.8) | 0.171 | 84.3 (61.9) | 90.5 (67.0) | 0.107 |
| Glucocorticoid dose converted to prednisolone equivalent | 12.26 (12.87) | 13.76 (13.53) |  | 12.03 (12.92) | 13.19 (13.26) |  |
| Immunosuppressant | 70 (74.5) | 23 (53.5) | 0.448 | 99.2 (72.8) | 87.8 (65.0) | 0.168 |
| Biologic immunosuppressive drugs | 28 (29.8) | 10 (23.3) | 0.148 | 37.7 (27.7) | 39.0 (28.9) | 0.026 |
| Antineoplastic drugs | 5 (5.3) | 7 (16.3) | 0.359 | 9.0 (6.6) | 15.5 (11.5) | 0.171 |
| Blood test findings |  |  |  |  |  |  |
| White blood cell count, /μL | 8724.68 (4808.22) | 8853.95 (5067.86) | 0.026 | 8979.85 (5131.52) | 9315.36 (5641.25) | 0.062 |
| Neutrophil count ^a^, /μL | 6579.19 (3295.39) | 6799.71 (3697.74) | 0.063 | 6653.96 (3279.26) | 6880.84 (3689.11) | 0.065 |
| Lymphocyte count ^a^, /μL | 1146.43 (1157.49) | 1173.61 (1376.02) | 0.021 | 1136.16 (1097.80) | 1387.02 (1822.38) | 0.167 |
| Hemoglobin, g/dL | 11.74 (1.96) | 11.36 (1.74) | 0.202 | 11.75 (1.94) | 11.39 (1.72) | 0.195 |
| Platelet count, ×10⁴/μL | 21.39 (10.11) | 22.11 (10.12) | 0.072 | 21.39 (9.98) | 22.66 (9.55) | 0.130 |
| Albumin, g/dL | 3.07 (0.60) | 2.94 (0.63) | 0.218 | 3.04 (0.60) | 3.07 (0.64) | 0.053 |
| Lactate dehydrogenase, IU/L | 400.01 (151.99) | 417.88 (164.33) | 0.113 | 408.42 (161.70) | 412.15 (151.47) | 0.024 |
| Serum sodium, mEq/L | 137.39 (3.68) | 137.86 (5.24) | 0.103 | 137.18 (3.62) | 137.42 (4.72) | 0.056 |
| Serum potassium, mEq/L | 4.29 (0.49) | 4.07 (0.45) | 0.463 | 4.29 (0.49) | 4.15 (0.40) | 0.304 |
| Creatinine, mg/dL | 1.12 (1.30) | 1.10 (1.82) | 0.011 | 1.11 (1.31) | 1.18 (1.92) | 0.046 |
| Creatinine clearance, mL/min | 57.85 (26.47) | 64.31 (29.30) | 0.231 | 59.24 (26.61) | 58.84 (27.05) | 0.015 |
| Altered consciousness | 1 (1.1) | 0 (0.0) | 0.147 | 2.2 (1.6) | 0.0 (0.0) | 0.180 |
| Hypotension (systolic pressure <90 mmHg) | 3 (3.2) | 0 (0.0) | 0.257 | 3.0 (2.2) | 0.0 (0.0) | 0.212 |
| Respiratory status |  |  | 0.251 |  |  | 0.136 |
| Without oxygen | 59 (62.8) | 23 (53.5) |  | 82.9 (60.8) | 86.7 (64.2) |  |
| Administration of oxygen | 34 (36.2) | 20 (46.5) |  | 52.4 (38.4) | 48.3 (35.8) |  |
| 1−4 L/min | 23 (24.5) | 10 (23.3) |  | 33.5 (24.6) | 23.9 (17.7) |  |
| 5−10 L/min | 4 (4.3) | 6 (14.0) |  | 5.9 (4.3) | 12.5 (9.3) |  |
| 11−15 L/min | 7 (7.4) | 4 (9.3) |  | 12.9 (9.5) | 11.9 (8.8) |  |
| Mechanical ventilation | 1 (1.1) | 0 (0.0) |  | 1.0 (0.7) | 0.0 (0.0) |  |
| Time from admission to treatment initiation | 0.13 (0.34) | 3.63 (1.45) | 3.331 | 0.14 (0.35) | 3.56 (1.49) | 3.151 |
| Initial therapeutic agents |  |  | 0.069 |  |  | 0.171 |
| SMX/TMP | 85 (90.4) | 38 (88.4) |  | 124.3 (91.2) | 115.9 (85.9) |  |
| Dose of SMX/TMP administration, mg/kg/d | 17.71 (13.55) | 15.64 (9.43) |  | 17.10 (12.59) | 17.62 (11.01) |  |
| Atovaquone | 7 (7.4) | 4 (9.3) |  | 9.4 (6.9) | 15.5 (11.5) |  |
| Pentamidine | 2 (2.1) | 1 (2.3) |  | 2.6 (1.9) | 3.5 (2.6) |  |
| Total treatment duration | 17.36 (6.91) | 19.86 (12.30) | 0.251 | 16.96 (7.08) | 18.94 (11.18) | 0.211 |
| Adjunctive glucocorticoid therapy |  |  |  |  |  |  |
| None | 10 (10.6) | 7 (16.3) | 0.166 | 13.6 (10.0) | 21.1 (15.6) | 0.170 |
| Yes (mild-to-moderate dose) | 29 (30.9) | 18 (41.9) | 0.230 | 43.8 (32.2) | 50.6 (37.5) | 0.112 |
| Yes (steroid pulse therapy) | 55 (58.5) | 18 (41.9) | 0.338 | 78.8 (57.9) | 63.3 (46.9) | 0.221 |

Continuous and categorical variables are expressed as the mean ± standard deviation and number (%), respectively

Adjusted totals and numbers represent the effective sample size and event counts after inverse probability weighting, where each patient contributes a fractional weight rather than a discrete count

^a^ Missing data were observed in one case each for neutrophil and lymphocyte counts

SMD, standardized mean difference, TMP-SMX, Trimethoprim-sulfamethoxazole

**Supplementary table 4.** Summary of clinical outcomes for the overall population in the sensitivity analysis using inverse probability of treatment weighting with truncation at the 1st and 99th percentiles

|  | **Unadjusted patient cohort** | | | **Adjusted patient cohort** | | |
| --- | --- | --- | --- | --- | --- | --- |
|  | **Early treatment group, n = 94** | **Late treatment group, n = 43** | ***P*** | **Early treatment group, n = 136.2** | **Late treatment group, n = 135.0** | ***P*** |
| Primary endpoint |  |  |  |  |  |  |
| 30-day mortality | 11 (11.7) | 5 (11.6) | 1.000 | 19.0 (14.0) | 11.1 (8.2) | 0.313 |
| Secondary |  |  |  |  |  |  |
| 180-day mortality | 18 (19.1) | 11 (25.6) | 0.529 | 29.3 (21.5) | 24.1 (17.8) | 0.610 |

Categorical variables are expressed as number (%).

Adjusted totals and numbers represent the effective sample size and event counts after inverse probability weighting, where each patient contributes a fractional weight rather than a discrete count

**Supplementary table 5.** Baseline characteristics of the subgroup requiring oxygen supplementation in the sensitivity analysis using inverse probability of treatment weighting with truncation at the 1st and 99th percentiles

|  | **Unadjusted patient cohort** | | | **Adjusted patient cohort** | | |
| --- | --- | --- | --- | --- | --- | --- |
|  | **Early treatment group, n = 35** | **Late treatment group, n = 20** | **SMD** | **Early treatment group, n = 52.38** | **Late treatment group, n = 49.60** | **SMD** |
| Age, years | 71.89 (9.48) | 72.60 (8.04) | 0.081 | 72.12 (9.55) | 72.54 (7.43) | 0.050 |
| Female | 16 (45.7) | 11 (55.0) | 0.187 | 23.8 (45.4) | 26.9 (54.2) | 0.176 |
| Weight, kg | 53.20 (10.92) | 55.25 (11.31) | 0.184 | 53.15 (10.79) | 54.79 (11.39) | 0.148 |
| Hospital |  |  |  |  |  |  |
| Kameda Medical Center | 27 (77.1) | 17 (85.0) | 0.469 | 42.0 (80.2) | 38.1 (76.9) | 0.207 |
| Seirei Hamamatsu General Hospital | 8 (22.9) | 2 (10.0) |  | 10.4 (19.8) | 10.5 (21.1) |  |
| Seirei Mikatahara General Hospital | 0 (0.0) | 1 (5.0) |  | 0.0 (0.0) | 1.0 (2.0) |  |
| Underlying diseases |  |  |  |  |  |  |
| Malignancy | 8 (22.9) | 8 (40.0) | 0.376 | 15.4 (29.4) | 14.5 (29.2) | 0.003 |
| Hematologic malignancy | 4 (11.4) | 3 (15.0) | 0.106 | 7.4 (14.1) | 4.9 (9.9) | 0.131 |
| Solid tumor | 4 (11.4) | 6 (30.0) | 0.471 | 8.0 (15.3) | 11.4 (22.9) | 0.196 |
| Connective tissue disease | 28 (80.0) | 9 (45.0) | 0.775 | 39.4 (75.2) | 28.4 (57.2) | 0.388 |
| Immunosuppressive agents used |  |  |  |  |  |  |
| Glucocorticoid | 26 (74.3) | 15 (75.0) | 0.016 | 37.9 (72.3) | 42.5 (85.7) | 0.333 |
| Glucocorticoid dose converted to prednisolone equivalent | 12.60 (14.71) | 13.54 (14.66) |  | 11.81 (13.57) | 14.59 (14.72) |  |
| Immunosuppressant | 23 (65.7) | 5 (25.0) | 0.896 | 32.8 (62.5) | 17.6 (35.6) | 0.561 |
| Biologic immunosuppressive drugs | 5 (14.3) | 1 (5.0) | 0.319 | 7.1 (13.6) | 2.0 (4.0) | 0.342 |
| Antineoplastic drugs | 4 (11.4) | 5 (25.0) | 0.357 | 7.9 (15.0) | 9.6 (19.3) | 0.114 |
| Blood test findings |  |  |  |  |  |  |
| White blood cell count ^a^, /μL | 9670.29 (5173.56) | 8754.50 (3879.33) | 0.200 | 10799.59 (6781.78) | 8977.44 (3768.01) | 0.332 |
| Neutrophil count, /μL | 7486.90 (3161.21) | 7139.82 (3078.48) | 0.111 | 7676.17 (3285.66) | 7375.66 (3071.64) | 0.094 |
| Lymphocyte count ^a^, /μL | 1015.71 (716.18) | 921.35 (556.70) | 0.147 | 1072.61 (743.18) | 967.91 (646.40) | 0.150 |
| Hemoglobin, g/dL | 11.91 (2.23) | 11.70 (1.23) | 0.114 | 11.79 (2.12) | 11.91 (1.24) | 0.068 |
| Platelet count (×10⁴/μL) | 22.59 (11.54) | 21.76 (9.54) | 0.078 | 22.35 (11.30) | 22.23 (10.34) | 0.011 |
| Albumin, g/dL | 2.80 (0.54) | 2.69 (0.56) | 0.216 | 2.77 (0.53) | 2.73 (0.52) | 0.071 |
| Lactate dehydrogenase, IU/L | 418.34 (166.91) | 467.85 (188.61) | 0.278 | 443.49 (189.98) | 450.94 (166.78) | 0.042 |
| Serum sodium, mEq/L | 137.26 (4.07) | 139.50 (6.53) | 0.412 | 136.96 (3.91) | 139.82 (5.30) | 0.614 |
| Serum potassium, mEq/L | 4.22 (0.50) | 3.98 (0.50) | 0.466 | 4.22 (0.53) | 4.13 (0.44) | 0.187 |
| Creatinine, mg/dL | 1.34 (1.76) | 0.78 (0.27) | 0.444 | 1.19 (1.59) | 0.80 (0.21) | 0.341 |
| Creatinine clearance, mL/min | 54.60 (26.19) | 68.98 (28.82) | 0.522 | 58.09 (26.20) | 62.82 (23.00) | 0.192 |
| Altered consciousness | 0 (0.0) | 0 (0.0) | <0.001 | 0 (0.0) | 0 (0.0) | <0.001 |
| Hypotension (systolic pressure <90 mmHg) | 2 (5.7) | 0 (0.0) | 0.348 | 2.0 (3.8) | 0.0 (0.0) | 0.282 |
| Respiratory status |  |  | 0.243 |  |  | 0.197 |
| Administration of oxygen | 34 (97.1) | 20 (100.0) |  | 51.4 (98.1) | 49.6 (100.0) |  |
| 1−4 L/min | 23 (65.7) | 10 (50.0) |  | 31.8 (60.8) | 21.5 (43.4) |  |
| 5−10 L/min | 4 (11.4) | 6 (30.0) |  | 5.5 (10.4) | 14.5 (29.2) |  |
| 11−15 L/min | 8 (22.9) | 4 (20.0) |  | 15.1 (28.8) | 13.6 (27.4) |  |
| Mechanical ventilation | 1 (2.9) | 0 (0.0) |  | 1.0 (1.9) | 0.0 (0.0) |  |
| Time from admission to treatment initiation | 0.11 (0.32) | 3.10 (1.12) | 3.625 | 0.11 (0.32) | 2.91 (1.09) | 3.475 |
| Initial therapeutic agents |  |  | 0.032 |  |  | 0.189 |
| SMX/TMP | 33 (94.3) | 19 (95.0) |  | 49.3 (94.2) | 44.1 (89.0) |  |
| Dose of SMX/TMP administration, mg/kg/d | 16.03 (7.21) | 12.49 (6.74) |  | 15.49 (6.87) | 13.74 (6.51) |  |
| Atovaquone | 2 (5.7) | 1 (5.0) |  | 3.0 (5.8) | 5.5 (11.0) |  |
| Total duration of treatment | 16.71 (8.65) | 20.15 (11.23) | 0.343 | 16.09 (8.64) | 21.58 (14.33) | 0.464 |
| Adjunctive glucocorticoid therapy |  |  |  |  |  |  |
| None | 1 (2.9) | 2 (10.0) | 0.294 | 1.1 (2.0) | 3.9 (7.8) | 0.270 |
| Yes (mild-to-moderate dose) | 15 (42.9) | 11 (55.0) | 0.245 | 22.9 (43.7) | 27.2 (54.8) | 0.224 |
| Yes (steroid pulse therapy) | 19 (54.3) | 7 (35.0) | 0.395 | 28.4 (54.3) | 18.6 (37.4) | 0.344 |

Continuous and categorical variables are expressed as the mean ± standard deviation and number (%), respectively.

Adjusted totals and numbers represent the effective sample size and event counts after inverse probability weighting, where each patient contributes a fractional weight rather than a discrete count

^a^ Missing data were observed in one case each for neutrophil and lymphocyte counts

SMD, standardized mean difference.

**Supplementary table 6.** Clinical outcomes of the subgroup requiring oxygen supplementation in the sensitivity analysis using inverse probability of treatment weighting with truncation at the 1st and 99th percentiles

|  | **Unadjusted patient cohort** | | | **Adjusted patient cohort** | | |
| --- | --- | --- | --- | --- | --- | --- |
| **Survival** | **Early treatment group, n = 35** | **Late treatment group, n = 20** | ***P*** | **Early treatment group, n = 52.4** | **Late treatment group, n = 49.6** | ***P*** |
| Primary endpoint |  |  |  |  |  |  |
| 30-day mortality | 7 (20.0) | 4 (20.0) | 1.000 | 13.0 (24.8) | 8.7 (17.6) | 0.578 |
| Secondary endpoint |  |  |  |  |  |  |
| 180-day mortality | 11 (31.4) | 9 (45.0) | 0.475 | 17.7 (33.9) | 19.8 (40.0) | 0.692 |

Categorical variables are expressed as number (%)

Adjusted totals and numbers represent the effective sample size and event counts after inverse probability weighting, where each patient contributes a fractional weight rather than a discrete count
